# The Use of Partial Textual Stimuli within an Interactive Task for Increasing Reports of Past Behavior with a Child with Autism

**DOI:** 10.1007/s40616-025-00218-w

**Published:** 2025-06-24

**Authors:** Masyn S. McWilliams, Robbie J. Hanson

**Affiliations:** College of Education and Human Services, Lindenwood University, 209 South Kingshighway, St. Charles, MO 63301 USA

**Keywords:** Autism, Past event, Textual stimuli, Verbal behavior

## Abstract

Individuals with autism spectrum disorder (ASD) may face challenges with reporting past behavior. Although some behavior-analytic studies have shown success with increasing these reports, the use of materials within activities that do not repeat has not been assessed (e.g., Shillingsburg et al., 2017). Thus, the purpose of the current study was to expand upon previous research (e.g., Shillingsburg et al. 2017; 2019) by utilizing novel materials within activities completed and examining the use of partial textual stimuli within an interactive task to increase reports of past behavior for one child with autism. The results showed an increase in reports of past behavior following intervention across three activities as well as an increase in varied responses.

Reporting past behavior will inevitably be required to maintain social interactions (e.g., “What did you do last weekend?”), when recounting safety information (e.g., “Where did you get this cut?”), or within academic environments (Keesey-Phelan et al., 2022). Previous behavior-analytic research has examined various strategies for improving reports of past behavior including teaching self-questioning and visual imagining (e.g., Keesey-Phelan et al., 2022), or increasing attending during the event, such as requiring tacting during the activity (e.g., Keesey-Phelan et al., 2024), among others. Shillingsburg et al. (2017) examined the use of prompts, fading, and differential reinforcement to increase past behavior reports for two children with autism. The researchers conducted immediate and end-of-day probes after the participants engaged in activities within specific locations (e.g., “What did you do in [location]?”). Following baseline, the researchers provided echoic or tact prompts for immediate probes. Additionally, the researchers implemented an error correction and fading procedure in which practice opportunities were provided in between immediate and end-of-the-day probes at 15 min intervals. The prompt and fading procedure, in combination with several other procedural modifications (e.g., end-of-day practice trials, immediate probe fading), resulted in both participants demonstrating correct responding for end-of-day probes after immediate probes were removed. Similarly, Shillingsburg et al. (2019) replicated these findings with three children with autism via gestural prompts (rather than echoic or tact prompts) who used speech generating devices.

Although these studies demonstrated promising results, the use of novel materials within activities were not assessed. Thus, the purpose of the current study was to expand upon previous research (e.g., Shillingsburg et al., 2017, 2019) by utilizing novel materials within activities completed and examine the use of partial textual stimuli within an interactive task to increase reports of past behavior for one child with autism.

## Method

### Participant and Setting

A 4-year-old Caucasian, English-speaking, female (referred to as Ellie hereafter) diagnosed with autism participated. She attended a behavioral treatment center before the start of her school day and her skills were assessed prior to the current study with the *Verbal Behavior Milestones Assessment and Placement Program* (VB-MAPP; Sundberg, 2008) with skills demonstrated at Level 2 and emerging at Level 3. At the time of the study, Ellie could read short novel sentences without assistance. The experimenter assessed assent prior to each session based on Ellie’s willingness to engage with the primary investigator. The study was conducted in instructional rooms normally used for behavior intervention. The room measured approximately 4.57 m by 3.66 m and included a table and chairs as well as various play items.

### Materials

**Fig. 1:**
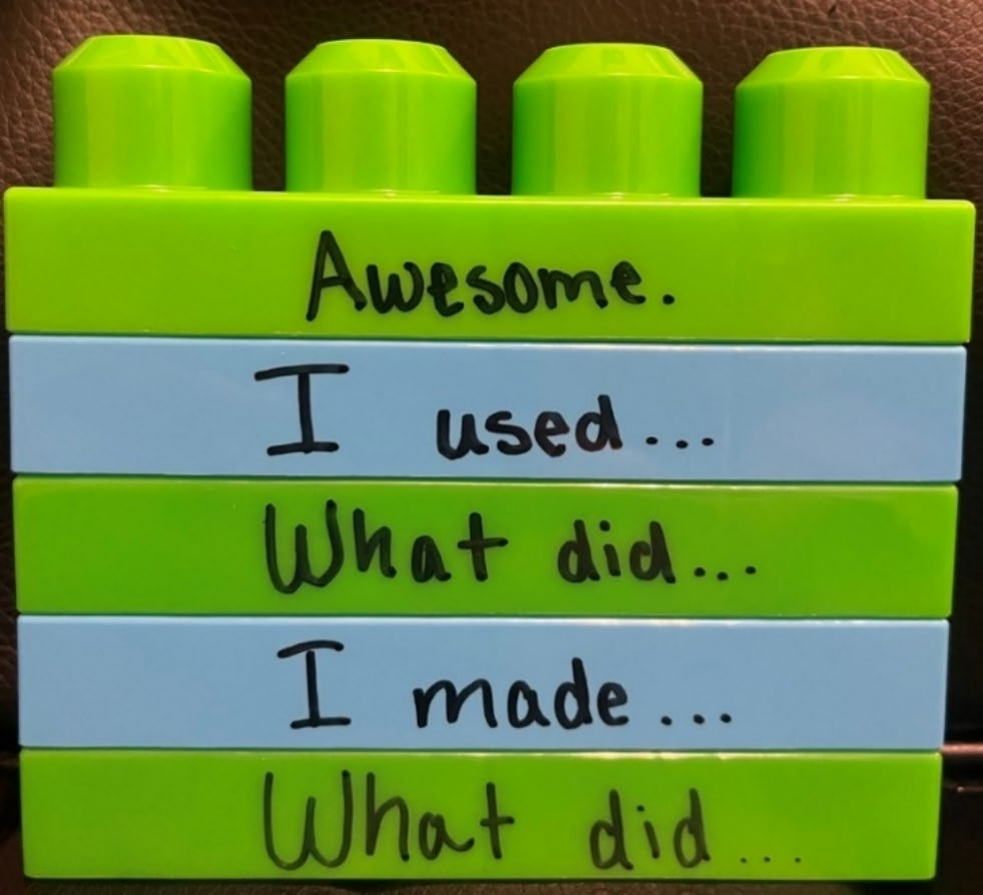
Stacking blocks example

The experimenter used three sets of textual and vocal stimuli, each specific to a different activity (i.e., book reading, craft, and gross motor room play). Each set consisted of five lines, including statements and questions for the experimenter and Ellie (three lines for the experimenter and two lines for Ellie). The lines were designed to allow for items within each activity to change across days (e.g., a different book read each day; see Table 1). A portion of each line was written on large stacking blocks (see Fig. 1) as partial textual stimuli with every other block being a different color when stacked (e.g., green blocks for experimenter, blue for Ellie).

**Table 1:** Textual and vocal stimuli used across activities

Activities	Textual/Visual Stimuli
Book	Experimenter: **What** book **did** you read today? Participant: **I read** _________________. (Insert book title) Experimenter: **Nice, who or what did** you read about? Participant: **I liked** _________________________. (Insert characters of book) Experimenter: That’s **awesome! Anything** else you want to tell me about the book?
Craft	Experimenter: **What did** you make during craft time today? Participant: **I made** _________. (describe the craft item) Experimenter: **How cool! What did** you use to make your craft? Participant: **I used** ________ (describe the colors used in the craft) Experimenter: **Awesome! Anything** else you want to share about your craft?
Gross Motor Room	Experimenter: **What did** you play with in the playroom today? Participant: **I played with** _______________. (describe activity or toys) Experimenter: **Awesome! Who did** you play with, or did you play alone? Participant: **I played** with ____________ (names or alone), **and** we _________ (mention interaction) Experimenter: That **sounds fun! Anything** else that you want to tell me about what you did in the playroom?

The bolded portions indicate the words that were written on the stacking blocks

### Dependent Variables and Response Definitions

The primary dependent variable was the number of correct reports of past behavior across conditions and activities. Correct reports were defined as vocal responses corresponding to the vocal stimulus presented (e.g., the question asked) and in alignment with the activity that occurred (see Table 2 for examples of correct and incorrect responses per activity). Responses were measured 1 h after the activity occurred (hereafter referred to as Opportunity 1) and then again approximately 20–40 min after the first opportunity (hereafter referred to Opportunity 2).

**Table 2:** Primary dependent variable examples

Activity	Examples of correct responses	Examples of incorrect responses
Book reading	Title of book (e.g., “*Froggy’s Sleepover*”; “*Five Little Monkeys Bake a Cake*”) Theme of book (e.g., “Froggy had a sleep over with his friends”) Specifics of material read (e.g., “…he forgot his sleeping bag and bathing suit”; “…they ate popcorn with flies”; “They had to race to the cake to stop it from burning”)	Incorrect title of book or title without enough information to determine if correct (e.g., “Froggy” is incorrect because there are multiple books with Froggy as the main character but with different themes and titles) Incorrect theme of book Specifics not part of what was read
Craft	Name of craft and/or materials used (e.g., “Butterfly”; “I made a kite with bows”)	Incorrect craft named Incorrect materials named Additional information not relevant to craft made
Gross motor	Peers played with and/or activities engaged in (e.g., “We stood like flamingos”; “Played ghosts with [peer’s name]”)	Incorrect peers identified Incorrect activities identified Additional information unrelated to gross motor room

Incorrect responses were defined as vocal responses that did not correspond to the vocal stimulus presented or the activity (see Table 2 for examples), no response, and responses in which an echoic prompt was required. Additional data were taken on the number of correct varying reports across conditions and activities during Opportunity 2. Correct varying reports were defined as vocal responses corresponding to the vocal stimulus presented and the activity but that were not identical to the report emitted during Opportunity 1 (e.g., if Ellie responded with, “I used pink and yellow paint to decorate the kite” a correct varying response during Opportunity 2 may be, “I put bows on the tail of the kite”).

### General Procedure

All activities were part of Ellie’s regular day, and she had no previous exposure to the specific materials used. The book activity occurred with one other child and the book was read to them by an adult (approximately 10 min). The craft was led by an adult (approximately 10 min), and the gross motor room activity occurred in the presence of four–seven other children (approximately 15 min). Activities were randomly rotated each day, and the same materials were never presented more than once. For each opportunity, all five lines for each set were presented with partial textual stimuli on stacking blocks and there were three possible correct responses for Ellie per set. Partial textual stimuli and a stacking block were not available to Ellie for the last possible correct response (e.g., after the experimenter stated, “Anything else you want to tell me about the book?”). Echoic prompts were implemented during intervention as part of the error correction. Ellie engaged in regularly scheduled programming in between activity completion and experimental sessions. A free operant preference assessment was conducted at the beginning of each day during intervention to identify highly preferred items.

#### Block Stacking Pretraining

During block stacking pretraining, the experimenter presented Ellie with stacking blocks unstacked in the middle of the table between the experimenter and Ellie. The blocks included partial textual stimuli for three general conversations unrelated to past behavior in which Ellie had previously demonstrated mastery. This condition was included to ensure that any incorrect responses observed during the intervention phase were not due to difficulty with block stacking. If Ellie did not read or read a particular line incorrectly, the experimenter provided an echoic prompt to evoke the textual prompt printed on the block. If Ellie attempted to stack a block before the experimenter had finished their turn, a vocal prompt was provided (e.g., “Remember, you need to wait until I’m finished”). Ellie was required to engage in 100% correct and independent responding across all three conversations to move on.

#### Baseline

During baseline, the experimenter called Ellie over to the table after a determined length of time past the completion of an activity. The book and craft activity were completed within the same room, whereas the gross motor activity was completed in a neighboring room. At the beginning of each trial, the experimenter presented the first line from one of the three sets. Regardless of correct or incorrect responding, the experimenter moved on to the next lines in the set until the last line was presented. Ellie was given 5 s to respond, and general acknowledgement was provided after each response (e.g., “Okay thanks”). No textual stimuli or stacking blocks were presented during baseline, and there were no additional prompts.

#### Intervention

Intervention was conducted similar to baseline, however, after Ellie was called to the table, the experimenter placed the blocks unstacked on the table in front of Ellie. The experimenter then placed the block with the first line on the table and presented the scripted question. Ellie was required to stack the block containing the partial textual stimuli related to the question and respond to the question. If Ellie responded incorrectly or did not respond within 5 s of the experimenter’s question, a vocal prompt (e.g., “It’s your turn”) to stack the block or an echoic prompt was delivered (to either read the textual stimuli for no responses or to prompt a correct response for incorrect responses). Descriptive praise was delivered for each correct response and 5-min access to highly preferred items was provided for correct responses to all questions in a set. Ellie was required to respond correctly for at least two of the three responses for Opportunity 1 across three consecutive days before being introduced to the next activity (i.e., craft, gross motor room).

### Experimental Design and Interobserver Agreement and Procedural Fidelity

A concurrent multiple baseline design across activities was implemented (Kazdin, 2021). Interobserver agreement (IOA) data were collected by a secondary observer for 50% of trials. IOA was calculated by dividing the number of agreements by the number of agreements and disagreements and multiplying by 100. IOA averaged 94.2% (range, 67–100%). Procedural fidelity (PF) data were collected by a secondary observer across conditions for 35% of trials. PF was calculated by dividing the number of correctly performed steps by the total steps and multiplying by 100. PF averaged 96.8% (range, 87.5–100%).

## Social Validity

To assess social validity of the intervention, a five-question survey was administered to the parents of Ellie within the same week that intervention concluded in which a rating scale was used ranging from 1 – *strongly disagree* to 5 – *strongly agree*.

## Results

**Fig. 2:**
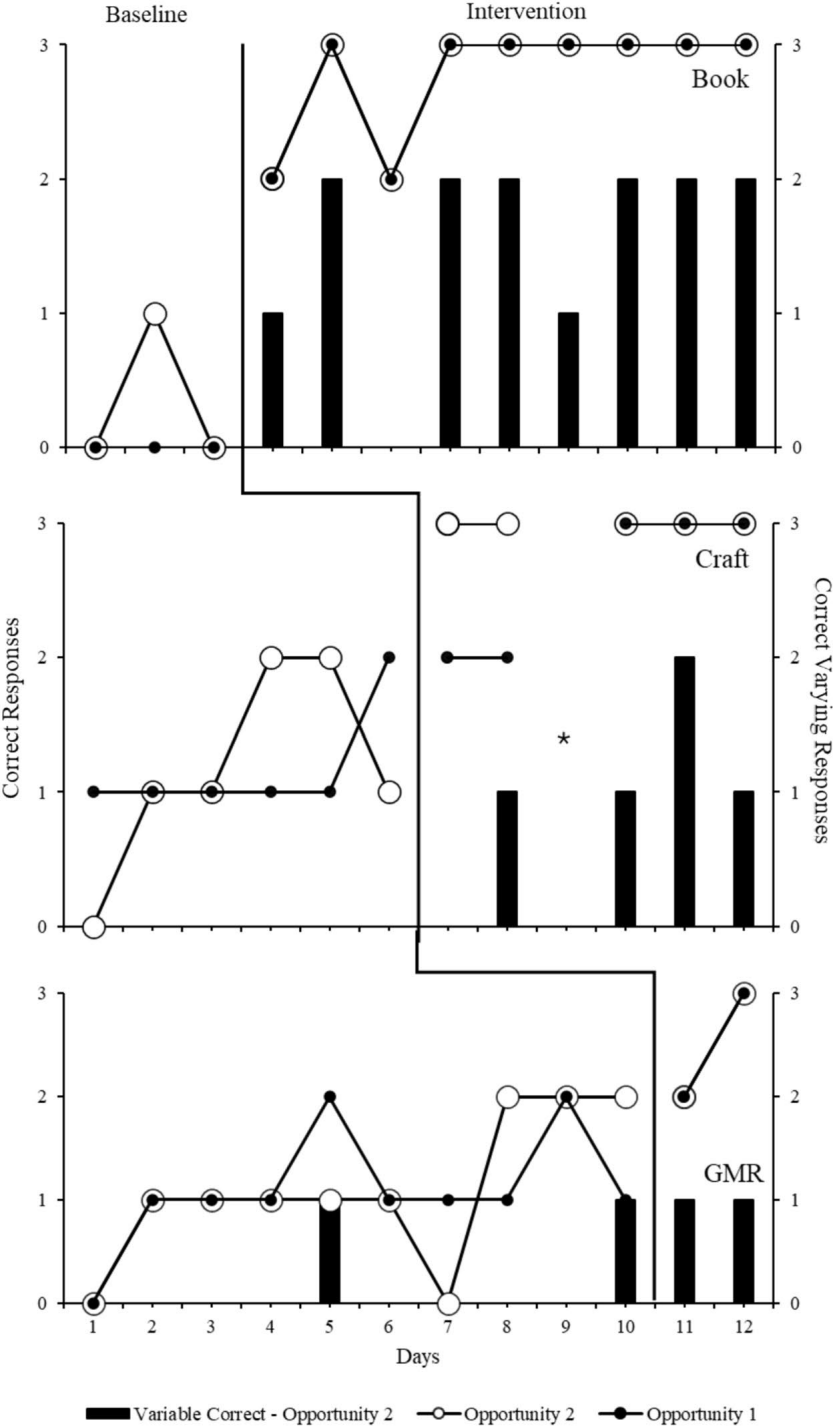
Correct responses across conditions and activities. *Note*. * participant requested to end the session after the first response; *GMR* gross motor room

Figure 2 shows the number of correct reports and number of correct varying reports across conditions and activities. During baseline for the book, Ellie emitted zero correct reports during Opportunity 1 and one correct report in total during Opportunity 2, with no correct varying reports. Ellie frequently responded with “I don’t know” during baseline or with a statement that did not align with the activity. Following intervention, Ellie emitted an average of 2.78 (range, 2–3) correct reports for both opportunities and an average of 1.56 (range, 0–2) correct varying reports. During baseline for the craft activity, Ellie averaged 1.16 (range, 1–2) correct reports for Opportunity 1, averaged 1.16 (range, 0–2) correct reports for Opportunity 2, and emitted zero correct varying reports. Following intervention, Ellie averaged 2.6 (range, 2–3) correct reports for Opportunity 1, averaged 3 (no range) correct reports for Opportunity 2, and averaged 1 (range, 0–2) correct varying report. It should be noted that data collected during Day 9 for the craft activity were not included in calculations as Ellie requested to end the activity after the first response. During baseline for the gross motor room, Ellie averaged 1.1 (range 0–2) correct reports during both opportunities and averaged 0.2 (range, 0–1) correct varying reports. During intervention, Ellie averaged 2.5 (range, 2–3) correct reports for both opportunities and averaged one correct varying report (no range).

The social validity assessment showed the parents strongly agreed that the target behaviors selected for the intervention were relevant, that they observed their child accurately report past behavior outside of those in the study, and that the overall experiment was a positive experience and that they felt well informed during the study. Additionally, they agreed that the intervention was effective in increasing both their child’s reciprocal conversation skills and reports of past behavior.

## Discussion

The purpose of the current study was to expand upon previous research (e.g., Shillingsburg et al., 2017, 2019) by utilizing novel materials within activities and examining the use of partial textual stimuli within an interactive task to increase reports of past behavior for one child with autism. The results showed that correct reports increased following intervention across both opportunities, as well as the number of correct varying reports across activities. Additionally, because the materials used during each activity never repeated and the same question–answer combinations were never taught and reinforced, responding was most likely under multiple sources of control (i.e., the question and additional stimuli generated by Ellie’s problem solving behaviors). These results are promising in that it demonstrated a potential procedure to evoke answers about past activities.

Although children with autism may display difficulties with reports of past behavior for which there is only one correct answer (e.g., personal information, preferences of peers; e.g., Fienup et al., 2013) and reports in which the answer changes contingent upon many variables (e.g., “What did you do last weekend?”), the sources of control differ. In the first circumstance in which there is only one correct answer, eventually the verbal discriminative stimulus will evoke the answer. However, in the second circumstance, problem solving is required as an ongoing skill and recall can be conceptualized, at least from a behavior-analytic perspective, as a problem to be solved (Miguel, 2018; Palmer, 1991; Skinner, 1957, 1984). Granted, although the question will typically exert some discriminative control over the answer, it is often the case that the question alone is insufficient to evoke the response and a sequence of precurrent behaviors must be emitted at either the overt or covert level (Palmer, 1991; Skinner, 1957). Further, when verbal discriminative stimuli fail to evoke problem solving behavior, supplemental stimuli (prompts, etc.) may be required (Palmer, 1991; Skinner, 1957). Thus, rather than teaching problem solving, the experimenters expanded upon previous research to explore the use of partial textual stimuli within an interactive activity as potential supplemental stimuli to evoke it. We chose to use textual stimuli as opposed to an echoic prompt as the former allows increased exposure to the stimulus. That is, the textual stimulus is not removed from the environment after presentation, whereas the echoic prompt, requiring an echoic response is removed after initial presentation (Farber & Dickson, 2023).

Although the current results are encouraging, several limitations should be noted. First, there appeared to be a potential carryover effect for the second activity after the intervention was introduced for the first activity. However, mastery criteria were not reached until the intervention was introduced for subsequent activities. Although increases in responding for other activities after the onset of intervention for only one may be advantageous in a clinical setting, it may pose threats to the internal validity of the study. Future research should consider replicating our procedures with other designs such as a multiple baseline across participants. Additionally, future research should examine expanding the number of possible responses across activities and the number of activities to prevent a possible ceiling effect.

Second, it remains unclear what combination of components (e.g., textual stimuli, interactive activity, reinforcement) during the intervention were responsible for behavior change. Future studies should attempt to isolate these variables. Third, it is unclear which behavioral mechanisms were responsible for the increase in responding during intervention. It is possible that when verbal discriminative stimuli were not sufficient to evoke reports of past behavior, the addition of supplemental stimuli served to evoke covert behavior in the form of an intraverbal chain or covert seeing, or some combination of the two. It is also possible that partial textual stimuli evoked the emission of an autoclitic frame which may have led to covert imagining and covert tacting. In other words, after stating, “I made…” Ellie covertly imagined the craft activity, the product of which served to evoke a tact (e.g., “kite with bows”) or a covert intraverbal chain to allow correct responding (degli Espinosa et al., 2021; Miguel, 2018). Future research should attempt to isolate the behavioral mechanisms during this type of intervention.

Next, only one participant was included in the current study. Therefore, it is unclear if similar results would be obtained with participants with similar or different characteristics, for reports of past behavior involving longer delays, and for different types of activities. Further, the stacking blocks and partial textual stimuli were never removed. Therefore, the extent to which the response would maintain in the absence of the blocks is unknown. This limitation speaks to the practicality of this intervention. However, the fact that textual stimuli were not provided for the last response in the conversation sequence, suggests that the intervention might be effective in evoking reports of past behavior. Future research should examine the complete removal of textual stimuli and the feasibility of implementing the intervention in other educational settings. Additionally, formalized pretesting for tacts of the materials and activities used was not completed and may have presented a limitation to the current study. However, this may be unlikely given the participant’s scores on the VB-MAPP.

Finally, the current study did not teach a problem-solving strategy but rather explored the use of supplemental stimuli to possibly evoke problem solving. Thus, future research should examine potential assessment measures to determine whether difficulties with reports of past behavior are (a) a function of deficits in attending; (b) influenced by the prompts or stimuli utilized; or (c) related to lack of problem solving skills. Despite these limitations, the results provide preliminary evidence for the use of partial textual stimuli within an interactive task to increase reports of past behavior for a child diagnosed with autism, and it is our hope that these results will encourage future research in this area.

## Data Availability

All data are available upon request.

## Declarations

### Author Note

This study is based on a thesis submitted by the first author under the supervision of the second author to the College of Education and Human Services at Lindenwood University in partial fulfillment of the requirements for an M.A. in Behavior Analysis.

All authors contributed to the study conception and design. Material preparation and data collection was performed by Masyn S. McWilliams, data analyses were performed by Masyn S. McWilliams and Robbie J. Hanson. The first draft of the manuscript was written by Masyn S. McWilliams and all authors commented on previous versions of the manuscript. All authors read and approved the final manuscript.

We would like to thank Caio F. Miguel for his valuable feedback on a previous version of this manuscript.
